# Transplantation of olfactory ensheathing cells on functional recovery and neuropathic pain after spinal cord injury; systematic review and meta-analysis

**DOI:** 10.1038/s41598-017-18754-4

**Published:** 2018-01-10

**Authors:** Babak Nakhjavan-Shahraki, Mahmoud Yousefifard, Vafa Rahimi-Movaghar, Masoud Baikpour, Farinaz Nasirinezhad, Saeed Safari, Mehdi Yaseri, Ali Moghadas Jafari, Parisa Ghelichkhani, Abbas Tafakhori, Mostafa Hosseini

**Affiliations:** 1 Sina Trauma and Surgery Research Center, Tehran University of Medical Sciences, Tehran, Iran; 2 Physiology Research Center and Department of Physiology, Faculty of Medicine, Iran University of Medical Sciences, Tehran, Iran; 3 Department of Medicine, School of Medicine, Tehran University of Medical Sciences, Tehran, Iran; 4 Department of Emergency Medicine, Shohadaye Tajrish Hospital, Shahid Beheshti University of Medical Sciences, Tehran, Iran; 5 Department of Epidemiology and Biostatistics, School of Public Health, Tehran University of Medical Sciences, Tehran, Iran; 6 Department of Emergency Medicine, School of Medicine, Bushehr University of Medical Sciences, Bushehr, Iran; 7 Department of Intensive Care Nursing, School of Nursing and Midwifery, Tehran University of Medical Sciences, Tehran, Iran; 8 Department of Neurology, School of Medicine, Imam Khomeini Hospital, Tehran University of Medical Sciences, Tehran, Iran; 9 Iranian Center of Neurological Research, Tehran University of Medical Sciences, Tehran, Iran; 10 Pediatric Chronic Kidney Disease Research Center, Tehran University of Medical Sciences, Tehran, Iran

## Abstract

There are considerable disagreements on the application of olfactory ensheathing cells (OEC) for spinal cord injury (SCI) rehabilitation. The present meta-analysis was designed to investigate the efficacy of OEC transplantation on motor function recovery and neuropathic pain alleviation in SCI animal models. Accordingly, all related studies were identified and included. Two independent researchers assessed the quality of the articles and summarized them by calculating standardized mean differences (SMD). OEC transplantation was shown to significantly improve functional recovery (SMD = 1.36; 95% confidence interval: 1.05–1.68; p < 0.001). The efficacy of this method was higher in thoracic injuries (SMD = 1.41; 95% confidence interval: 1.08–1.74; p < 0.001) and allogeneic transplants (SMD = 1.53; 95% confidence interval: 1.15–1.90; p < 0.001). OEC transplantation had no considerable effects on the improvement of hyperalgesia (SMD = −0.095; 95% confidence interval: −0.42–0.23; p = 0.57) but when the analyses were limited to studies with follow-up ≥8 weeks, it was associated with increased hyperalgesia (SMD = −0.66; 95% confidence interval: −1.28–0.04; p = 0.04). OEC transplantation did not affect SCI-induced allodynia (SMD = 0.54; 95% confidence interval: −0.80–1.87; p = 0.43). Our findings showed that OEC transplantation can significantly improve motor function post-SCI, but it has no effect on allodynia and might lead to relative aggravation of hyperalgesia.

## Introduction

Spinal cord injury (SCI) is among the most important causes of mortality and disability in the young, with a reported global prevalence of 236 to 4178 cases per one million people, to which 180,000 cases are added every year^1^. Nevertheless, no definite treatment has been introduced for SCI and most measures are supportive and aim at alleviating symptoms of the patients^2,3^. Functional impairment, neuropathic pain, and diminished quality of life are the most prominent complications that patients with SCI encounter^4^.

In recent years, regenerative medicine has opened a promising window towards effective treatments for SCI^5,6^. Cell therapy is one of the important methods applied in this field, and can improve symptoms associated with SCI through creating new neural connections at the level of injury and driving differentiation of cells into neurons along with its neuroprotective activities. Various sources can be used for cell therapy ranging from stem cells to neural supporting cells^7–11^. Olfactory ensheathing cells (OEC) are also viable candidates for cell therapy which can improve neuropathic pain and motor function in patients with SCI through multiple mechanisms including phagocytosis of axonal debris, immunoprotective characteristics that help axonal recovery, migration towards glial scars, and secretion of neutrotrophic factors^12^. However, their efficacy has been questioned by multiple surveys^13–16^ and their association with aggravation of allodynia and hyperalgesia has limited application of this method^17,18^. Moreover, only a few studies have assessed improvement in sensory function after OEC transplantation and they have reported contradictory results^19–21^.

In this regard, in 2014 a meta-analysis evaluated the efficacy of OECs on motor function recovery. Six studies were reviewed and the results depicted that transplantation of these cells can enhance functional recovery, but the study had considerable limitations. Firstly, in their systematic search only 95 non-repetitive articles were found. Secondly, their study suffered publication bias and their applied keywords were not able to yield the maximum number of articles^22^. In another meta-analysis conducted in 2016, OEC transplantation was shown to improve motor function of the animals with spinal cord injuries^23^, but the sensory status after transplantation was not evaluated in their study.

Therefore, a new meta-analysis was be performed with the same goal in order to reach a consensus. Furthermore, various treatment protocols have been used for OEC transplantation in spinal cord injuries that differed in injury phases, number of transplanted cells, OEC source (olfactory bulb or mucosa), timing of intervention, location of injury, use of antibiotic and immunosuppressive agents. These differences can cause significant variations in the reported results and the effect of treatment protocol on the efficacy of OEC transplantation in SCI is yet to be determined. Accordingly, the present systematic review and meta-analysis was designed to investigate the efficacy of this treatment along with the effects of different treatment protocols applied.

## Results

### Characteristics

**Figure 1:**
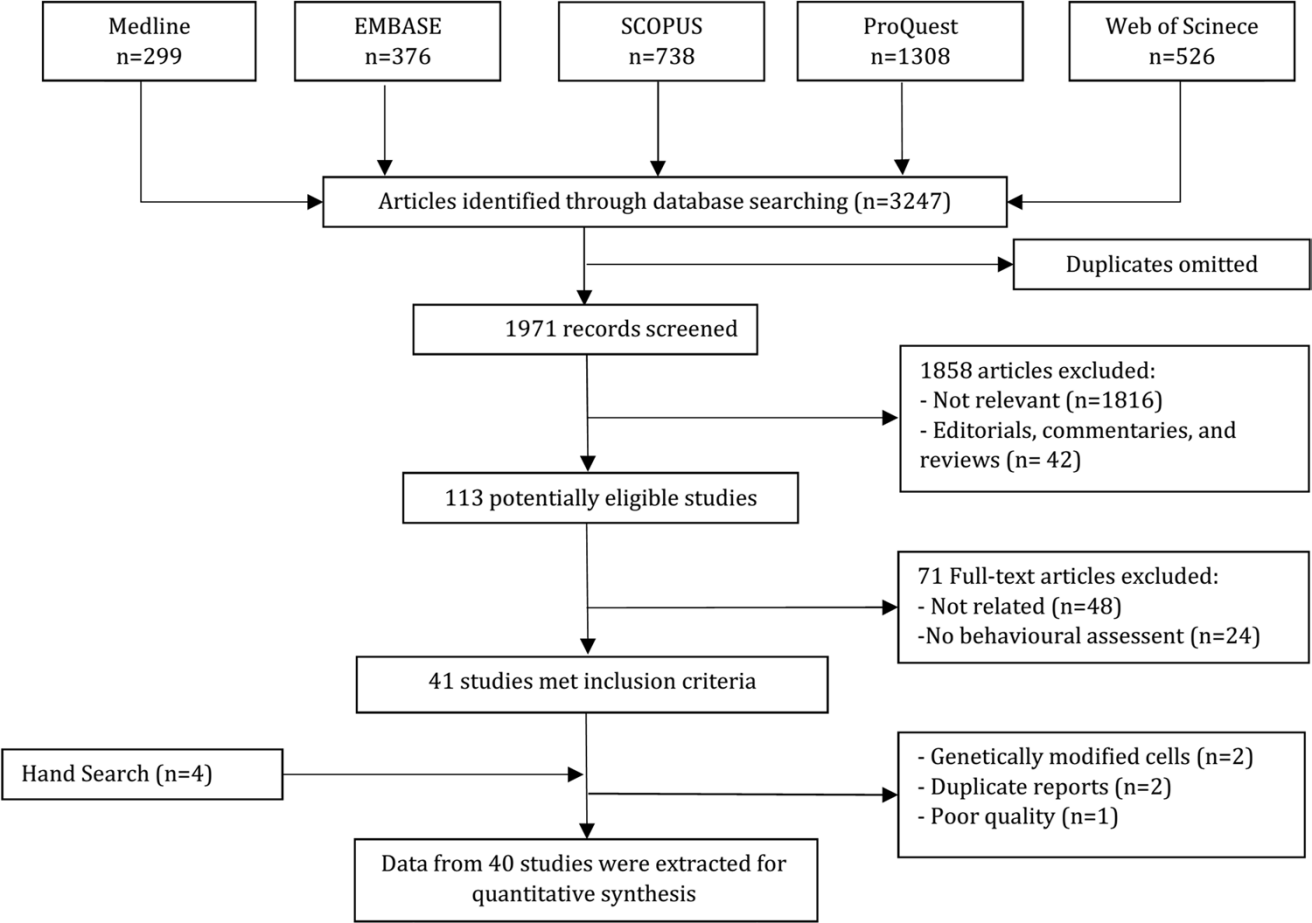
Flowchart of including studies in the meta-analysis.

Extensive search in databases produced 3247 articles, from which 1971 were found to be non-repetitive. A total of 113 articles were screened initially through evaluation of titles and abstracts, among which 41 met the inclusion criteria. Four additional studies were found through manual search. After elimination of duplicate reports and quality assessment of the articles, 40 studies were included in the meta-analysis^5,16,19–21,24–57^. Only three of these articles were Chinese language^32,53,56^ and the remaining 37 were in English^5,16,19–21,24–31,33–52,54,55,57^. Figure 1 depicts the flowchart drawn for the process of searching and selecting the articles.

Thirty-one articles only assessed motor function in the animals^5,16,24,26,27,30–32,34–39,41–49,51–57^, three evaluated sensory function^33,40,50^ and six included both these entities^19–21,25,28,29^. As presented in Table 1, showing the characteristics of included surveys, five studies reported at least two separate experiments; two compared the efficacy of transplantation in acute phase with the subacute phase^21,37^, one compared the efficacy in two injury models of transection and photochemical^37^, another article compared the efficacy of OECs obtained from the olfactory mucosa with those derived from the olfactory bulb on post-SCI motor function^53^ and in the last one the effects of allogeneic transplantation of OECs was compared with xenogeneic transplantation of these cells on neuropathic pain^33^. Accordingly, data from 45 experiments were extracted from these 40 articles.

**Table 1:** Characteristics of included studies.

Author, Year	Gender, Species, Weight (gr)	Model, Location of injury, Severity	OEC derivation origin, Donor, Graft, Dose, Type, Intervention time (day)	Immunosuppressive, Antibiotic, Blinding	Follow up (day)
Amemori^19^	Male, Rat, 270–300	Compression, T10, Moderate	Mucosa, Rat, IS, 3 × 105, Allogeneic, 7	Yes, Yes, Yes	56
Barbour^24^	Female, Rat, 180–200	Contusion, T10, Moderate	Bulb, Rat, IS, 5 × 105, Allogeneic, 14	No, Yes, Yes	126
Bretzner^25^	Male, Rat, 300–400	Contusion, C4–C5, Moderate	Mucosa, Mice, IS, 1.65 × 105, Xenogeneic, 1	Yes, No, No	28
Cao^26^	Female, Rat, 180–220	Transection, T8, Severe	Bulb, Rat, IS, 2 × 105, Allogeneic, 1	No, Yes, No	56
Deng^27^	Female, Rat, 240–270	Contusion, T10, Moderate	Bulb, Human, IS, 2.5 × 105, Xenogeneic, 1	Yes, Yes, Yes	35
Deumens^28^	Male, Rat, 200–250	Hemisection, T11, Severe	Bulb, Rat, IS, 4 × 105, Allogeneic, 1	No, No, Yes	70
Deumens^29^	Female, Rat, 185–220	Hemisection, T13, Severe	Bulb, Rat, IS, 4 × 105, Allogeneic, 1	No, No, No	70
Garcia–Alias^20^	Female, Rat, 200–250	Photochemical, T8, Moderate	Bulb, Rat, IS, 1.8 × 105, Allogeneic, 1	No, No, Yes	90
Gorrie^30^	Female, Rat, 110–147	Contusion, T10, Moderate	Mucosa, Human, IS, 1 × 106, Xenogeneic, 7	No, Yes, Yes	35
Guest^31^	Female, Rat, 140–155	Transection, T9–T10, Severe	Bulb, Macaca, IS, 4 × 105, Xenogeneic, 1	No, Yes, No	140
Jiang^32^	Male, Rat, 250−	Transection, T9, Severe	Bulb, Rat, IS, 1 × 105, Allogeneic, 1	No, No, No	84
Lang^33^	NR, Rat, 180–250	Hemisection, T10, Severe	Bulb, Mice, IS, 3 × 106, Xenogeneic, 1	Yes, No, No	28
Li^34^	Male, Rat, 200–250	Contusion, T10, Moderate	Bulb, Rat, IS, 9 × 104, Allogeneic, 7	No, Yes, No	42
Li^35^	Male, Rat, 200–250	Contusion, T10, Moderate	Bulb, Rat, IS, 9 × 104, Allogeneic, 7	No, Yes, Yes	36
Liu^36^	Both, Rat, 250–280	Hemisection, T13, Severe	Bulb, Rat, IT, 1 × 105, Allogeneic, 0.5	Yes, Yes, Yes	28
Luo^40^	Female, Rat, 180–250	Transection, T7–T9, Severe	Bulb, Rat, IS, 3 × 105, Allogeneic, 1	Yes, No, Yes	28
Lopez-Vales^37^	Female, Rat, 250–300	Transection, T8, Severe	Bulb, Rat, IS, 1.5 × 106, Allogeneic, 1 and 7	No, No, No	270
Lopez-Vales^37^	Female, Rat, 250–300	Transection and Photochemical, T8, Severe and Moderate	Bulb, Rat, IS, 1.5 × 106 and 1.8 × 105, Allogeneic, 1	No, No, No	90 and 270
Lopez-Vales^38^	Female, Rat, 250–300	Transection, T8, Severe	Bulb, Rat, IS, 1.5 × 106, Allogeneic, 45	No, No, No	195
Lu^39^	Female, Rat, 250–300	Transection, T10, Severe	Mucosa, Rat, IS, 1 × 105, Allogeneic, 28	No, Yes, Yes	70
Ma^41^	Female, Rat, 180–220	Contusion, T9, Moderate	Bulb, Rat, IS, 1 × 105, Allogeneic, 1	No, Yes, No	64
Masgutova^42^	NR, Rat, 200–250	Hemisection, T8, Severe	Mucosa, Human, IS, 2 × 105, Xenogeneic, 1	No, No, No	54
Pearse^43^	Female, Rat, 180–200	Contusion, T9, Moderate	Bulb, Rat, IS, 2 × 106, Allogeneic, 7	No, Yes, Yes	63
Resnick^44^	Male, Rat, 275–325	Contusion, T8–T9, Moderate	Bulb, Rat, IS, 2.5 × 105, Allogeneic, 1	No, No, Yes	42
Ruitenberg^45^	Female, Rat, 200	Hemisection, Cervical, Severe	Bulb, Rat, IS, 2 × 105, Allogeneic, 1	No, No, Yes	112
Salehi^46^	Female, Rat, 250–300	Compression, T8–T9, Moderate	Bulb, Rat, IS, 1 × 106, Allogeneic, 9	Yes, Yes, No	28
Sasaki^48^	Female, Rat, 150–179	Transection, T9, Severe	Bulb, Rat, IS, 1.5 × 105, Allogeneic, 1	No, No, Yes	35
Sasaki^47^	Female, Rat, 150–179	Transection, T9, Severe	Bulb, Rat, IS, 1.5 × 105, Allogeneic, 1	No, No, Yes	35
Sun^49^	Female, Rat, 220–250	Contusion, T10, Moderate	Bulb, Rat, IS, 4 × 105, Allogeneic, 14	No, Yes, Yes	63
Takami^16^	Female, Rat, 160–180	Contusion, T9, Moderate	Bulb, Rat, IS, 2 × 106, Allogeneic, 7	No, No, Yes	70
Takeoka^50^	Female, Rat, 210–250	Transection, T9, Sever	Bulb, Rat, IS, 4 × 105, Allogeneic, 1	No, No, Yes	210
Torres-Espin^21^	Female, Rat, 250–300	Contusion, T8–T9, Moderate	Bulb, Rat, IS, 4.5 × 105, Allogeneic, 1 and 7	No, Yes, No	35 and 42
Verdu^51^	Female, Rat, 250–300	Photochemical, T8, Moderate	Bulb, Rat, IS, 1.8 × 105, Allogeneic, 1	No, No, No	89
Wang^52^	Male, Rat, 200–250	Hemisection, T9, Severe	Bulb, Rat, IS, 1 × 107, Allogeneic, 7	No, No, Yes	77
Wang^53^	Male, mice, 28–30	Transection, T9–T11, Severe	Bulb and Mucosa, Rat, IS, 1 × 106, Allogeneic, 1	No, Yes, No	56
Wu^54^	NR, Rat, 200–240	Contusion, T9–T10, Moderate	Bulb, Rat, IS, 4 × 105, Allogeneic, 1	No, No, Yes	84
Wu^55^	Female, Rat, 250–300	Contusion, T10, Moderate	Bulb, Rat, IS, 3 × 105, Allogeneic, 7	No, Yes, Yes	28
Yazdani^5^	Female, Rat, 300–350	Contusion, T10, Moderate	Bulb, Rat, IS, 1 × 106, Allogeneic, 7	No, Yes, Yes	35
Yin^56^	Male, Rat, 250–300	Transection, T10, Severe	Bulb, Human, IS, 2.5 × 105, Xenogeneic, 10	No, No, No	70
Zhang^57^	Male, Rat, 200–250	Contusion, T10, Moderate	Bulb, Rat, IS, 6 × 105, Allogeneic, 7	Yes, Yes, Yes	63

IS: intra-spinal; IT: intrathecal; T: thoracic level of spinal cord; C: cervical level spinal cord.

Overall, data from 933 animals (control group = 464, treatment group = 469) were pooled together and analyzed. Thirty-one experiments were conducted on female animals and 14 were carried out on male subjects. Forty-three included rats and the other two evaluated mice. The most common injury models in the included articles were contusion with 19 experiments followed by transection with 14, hemisection with 7, photochemical with 3 and compression with 2 experiments. The mean (standard deviation) time interval between injury induction and transplantation was 5.3 ± 8.0 days (ranging from 0.05 to 45 days). In 26 experiments transplantation was done simultaneously with induction of injury (acute phase), in 15 experiments there was a 3 to 10-day gap between them (subacute phase) and in 4 experiments transplantation was carried out more than 2 weeks after the injury (chronic phase). Transplantation was intra-spinal in 44 experiments. Thirty-seven experiments used allogeneic transplants and the rest of studies applied xenogeneic transplantations. The number of transplanted cells per kg of body weight ranged from 3.6 × 10^5^ to 4.4 × 10^7^.

### Meta-analysis

#### Efficacy of OEC transplantation on functional improvement

**Figure 2:**
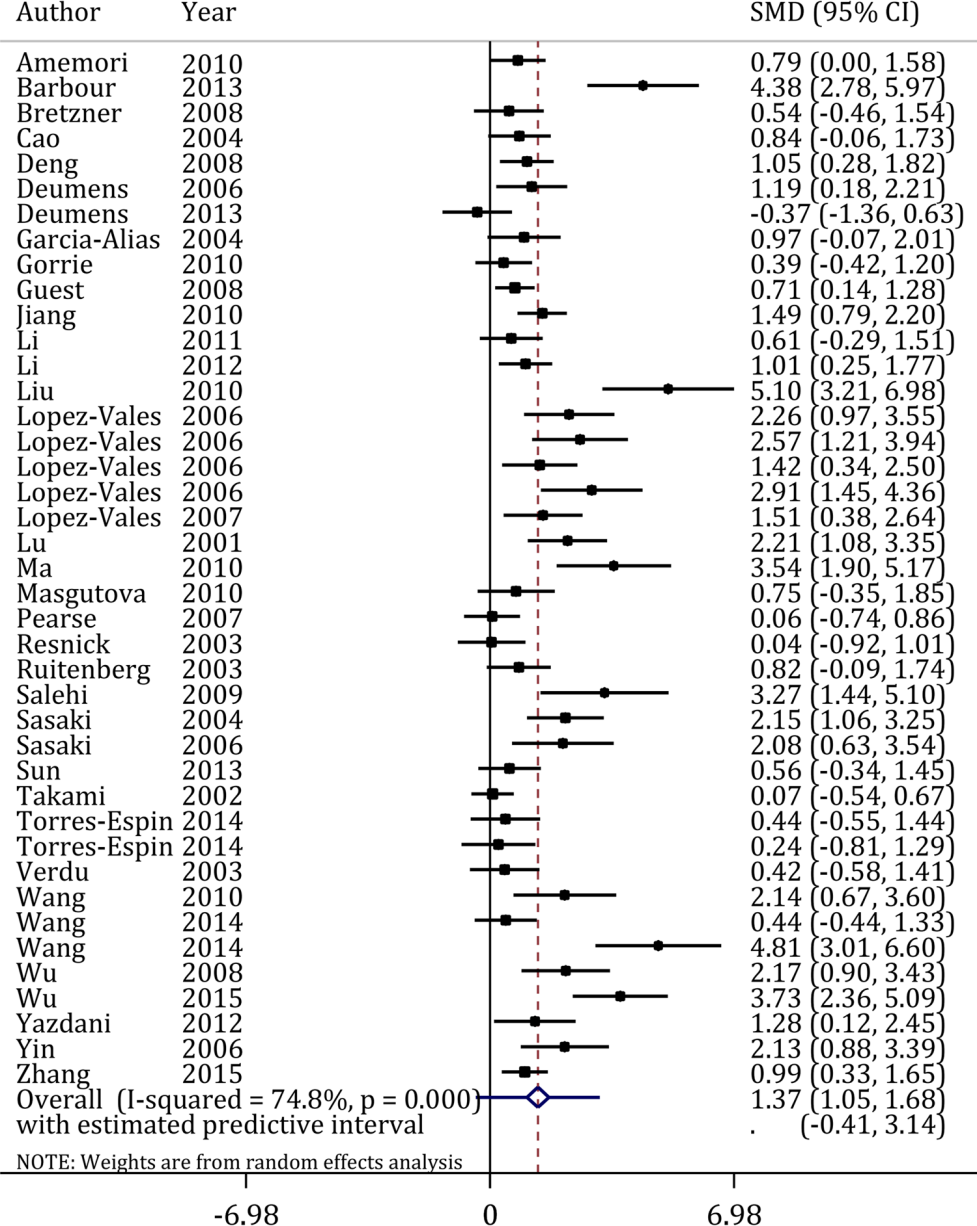
Efficacy of olfactory ensheathing cells transplantation on motor function recovery after spinal cord injury. CI: Confidence interval; SMD: Standardized mean difference.

37 articles^5,16,19–21,24–32,34–39,41–49,51–57^ including 41 experiments assessed the efficacy of OEC transplantation with different treatment protocols on the motor function recovery after SCI (Fig. 2). The results showed that OEC transplantation significantly improved functional recovery (Pooled SMD = 1.36; 95% confidence interval: 1.05–1.68; p < 0.001; I^2^ = 74.80%). This section of the analyses had no publication bias (Coefficient = 0.43; 95% confidence interval: −0.05–0.91 p = 0.08).

A significant heterogeneity was observed considering the effects of OEC transplantation on functional recovery (I^2^ = 74.80%; p < 0.001). Subgroup analysis revealed that differences in location of injury, graft type and donor species were the most prominent sources of heterogeneity between the studies. The results of these analyses indicated that the efficacy of OEC transplantation on motor function recovery is higher when the injury affects thoracic region (SMD = 1.41; 95% confidence interval: 1.08–1.74; p < 0.001) compared to cervical spinal injuries (SMD = 0.69; 95% confidence interval: 0.02–1.37; p = 0.045). Allogeneic transplant was also found to have a greater efficacy (SMD = 1.53; 95% confidence interval: 1.15–1.90; p < 0.001) compared to xenogeneic transplant (SMD = 0.82; 95% confidence interval: 0.44–1.20; p < 0.001). Transplantation of OECs acquired from rats provided a higher efficacy as well (SMD = 1.48; 95% confidence interval: 1.11–1.85; p < 0.001) (Table 2).

**Table 2:** Subgroup analyses of the effect of olfactory ensheathing cells on motor function recovery.

Characteristic	P for bias^a^	Model	P (I^2^)^b^	Effect Size^c^ (95% CI)	Predictive interval	P
**Gender**						
Male	0.41	REM	<0.001 (77.6%)	1.41 (0.82–2.01)	−0.69–3.52	<0.001
Female	0.03	REM	<0.001 (75.3%)	1.41 (1.02–1.80)	−0.48–3.19	<0.001
*Overall significance test among subgroups*						0.96
**Recipient species**						
Rat	0.08	REM	<0.001 (73.7%)	1.36 (1.05–1.68)	−0.36–3.00	<0.001
Mice	0.99	REM	<0.001 (94.5%)	2.53 (−1.72–6.82)	−0.30–0.33	0.24
*Overall significance test among subgroups*						0.41
**Injury model**						
Contusion	0.38	REM	<0.001 (76.7%)	1.07 (0.60–1.54)	−0.80–2.95	<0.001
Clip compression	0.99	REM	0.02 (83.2%)	1.89 (−0.52–4.30)	−0.62–5.30	0.12
Photochemical	0.99	REM	0.04 (68.5%)	1.24 (0.08–2.39)	−11.82–14.30	0.04
Hemisection	0.80	REM	<0.001 (84.0%)	1.70 (0.47–2.94)	−2.41–527	0.007
Transection	0.06	REM	<0.001 (67.8%)	1.74 (1.23–2.25)	0.00–3.48	<0.001
*Overall significance test among subgroups*						0.36
**Location of injury**						
Cervical	0.08	FEM	0.68 (0.0%)	0.69 (0.02–1.37)	NA	0.045
Thoracic	0.99	REM	<0.001 (75.9%)	1.41 (1.08–1.74)	−0.42–3.24	<0.001
*Overall significance test among subgroups*						0.40
**Severity of injury**						
Moderate	0.36	REM	<0.001 (74.5%)	1.14 (0.73–1.55)	−0.63–2.91	<0.001
Severe	0.05	REM	<0.001 (73.8%)	1.72 (1.24–2.21)	−0.41–3.14	<0.001
*Overall significance test among subgroups*						0.15
**OEC derivation origin**						
Bulb	0.09	REM	<0.001 (75.3%)	1.42 (1.07–1.76)	−0.41–3.14	<0.001
Mucosa	0.91	REM	<0.001 (80.0%)	1.38 (0.44–2.33)	−1.79–4.56	0.004
*Overall significance test among subgroups*						0.91
**Intervention phase** ^**d**^						
Acute	0.05	REM	<0.001 (74.2%)	1.42 (0.99–1.85)	−0.46–3.16	<0.001
Subacute	0.75	REM	<0.001 (75.1%)	1.21 (0.70–1.73)	−0.46–3.10	<0.001
Chronic	0.99	REM	<0.001 (83.3%)	2.06 (0.65–3.47)	−4.36–8.48	0.004
*Overall significance test among subgroups*						0.72
**Graft type**						
Allogeneic	0.81	REM	<0.001 (77.8%)	1.53 (1.15–1.90)	−0.50–3.46	<0.001
Xenogeneic	0.03	FEM	0.30 (18.0%)	0.82 (0.44–1.20)	NA	<0.001
*Overall significance test among subgroups*						0.24
**Number of transplanted cells**						
<3 × 10^6^ cell dose/kg	0.07	REM	<0.001 (73.6%)	1.37 (1.01–1.73)	−0.37–3.01	<0.001
≥3 × 10^6^ cell dose/kg	0.68	REM	<0.001 (80.1%)	1.52 (0.83–2.21)	−0.98–4.02	<0.001
*Overall significance test among subgroups*						0.82
**Donor species**						
Rat	0.81	REM	<0.001 (77.8%)	1.48 (1.11–1.85)	−0.50–3.46	<0.001
Human	0.29	FEM	0.14 (44.9%)	0.98 (0.34–1.62)	NA	0.008
Other	0.99	FEM	0.77 (0.0%)	0.67 (0.18–1.16)	NA	0.003
*Overall significance test among subgroups*						0.22
**Use of antibiotic**						
No	0.02	REM	<0.001 (68.0%)	1.34 (0.92–1.76)	−0.34–2.87	<0.001
Yes	0.98	REM	<0.001 (80.6%)	1.48 (1.00–1.97)	−0.63–3.60	<0.001
*Overall significance test among subgroups*						0.74
**Use of immunosuppressive agents**						
No	0.89	REM	<0.001 (79.7%)	1.38 (1.03–1.73)	−0.47–3.13	<0.001
Yes	0.05	REM	<0.001 (75.4%)	1.62 (0.72–2.52)	−1.34–4.57	<0.001
			*Overall significance test among subgroups*			0.66
**Blinding of observer**						
No	0.97	REM	<0.001 (72.8%)	1.34 (0.89–1.79)	−0.52–3.20	<0.001
Yes	0.007	REM	<0.001 (78.5%)	1.47 (1.01–1.94)	−0.55–3.34	<0.001
*Overall significance test among subgroups*						0.77
**Follow up period**						
<8 weeks	0.86	REM	<0.001 (75.4%)	1.32 (0.77–1.88)	−0.42–3.21	<0.001
≥8 weeks	0.07	REM	<0.001 (76.3%)	1.46 (1.06–1.86)	−0.77–3.42	<0.001
*Overall significance test among subgroups*						0.73

^a^Publication bias based on Begg’s and Egger’s test; ^b^Heterogeneity among studies; ^c^Standardized mean difference; ^d^Acute: immediately after injury, Subacute: 2–10 days after injury; Chronic: equal or more than 14 days. NA: Not applicable; REM: random effect model; FEM: fixed effect, CI: confidence interval

#### Efficacy of OEC transplantation on spinal cord injury induced hyperalgesia

**Figure 3:**
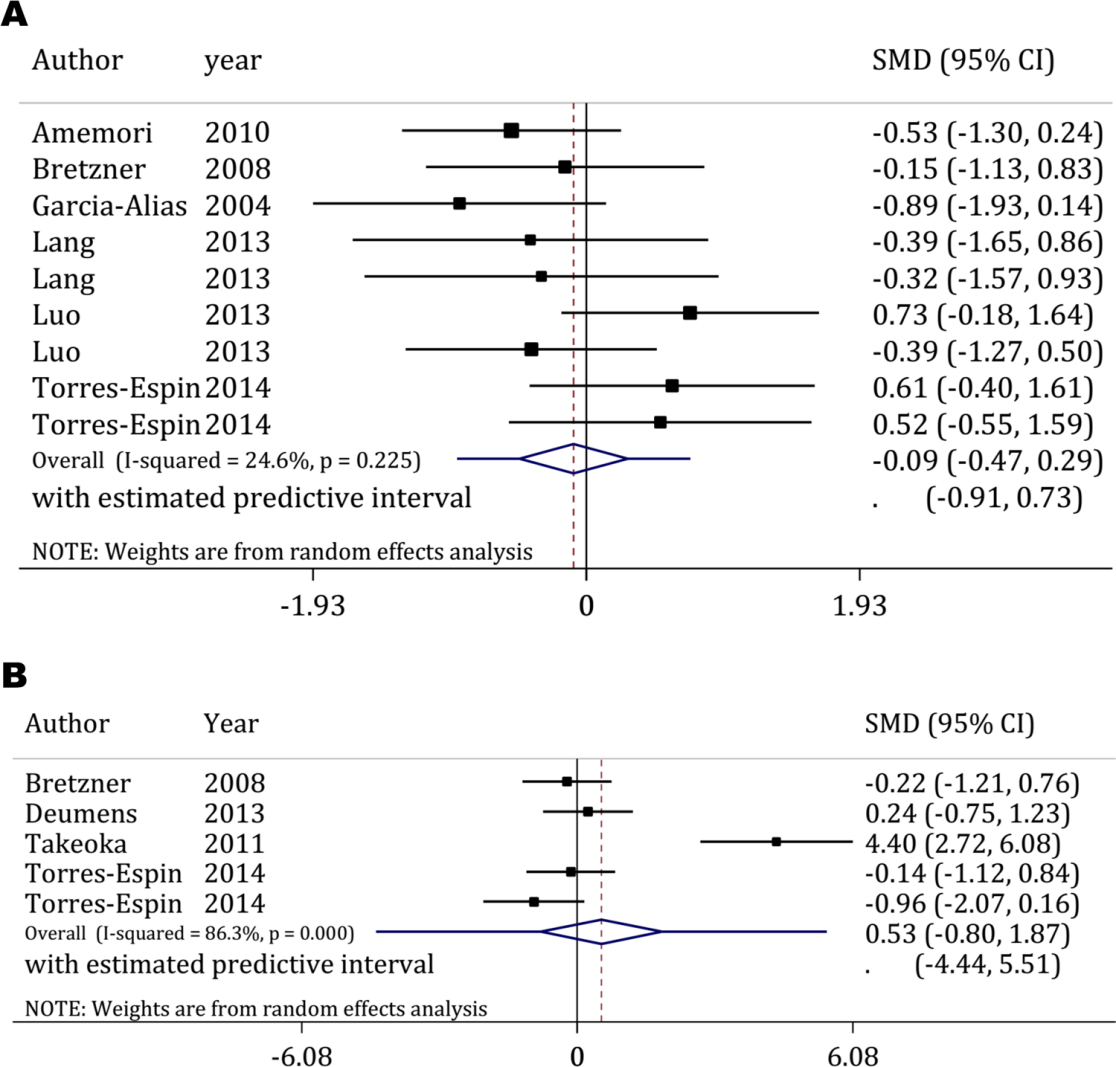
Efficacy of olfactory ensheathing cells transplantation on hyperalgesia (**A**) and allodynia (**B**) after spinal cord injury. CI: Confidence interval; SMD: Standardized mean difference.

Six articles^19–21,25,33,40^ including 9 experiments investigated the efficacy of OEC transplantation on improvement of hyperalgesia caused by SCI. As presented in Fig. 3, OEC transplantation showed no significant effects on improvement of hyperalgesia in animals post-SCI (Pooled SMD = −0.095; 95% confidence interval: −0.42–0.23; p = 0.57; I^2^ = 24.60%). No publication bias was present in this section of the analyses (Coefficient = 0.48; 95% confidence interval: −6.12–7.09 p = 0.87).

Nevertheless, the results of subgroup analysis showed that follow-up duration is a factor that affects the findings of the studies. OEC transplantation was found to aggravate hyperalgesia when only studies with follow-ups of equal or greater than 8 weeks were included (SMD = −0.66; 95% confidence interval: −1.28–0.04; p = 0.04), while analysis of the studies with shorter follow-ups found no significant relation between OEC transplantation and hyperalgesia (SMD = 0.13; 95% confidence interval: −0.26–0.51; p00.52) (Table 3).

**Table 3:** Subgroup analyses of the effect of olfactory ensheathing cells on hyperalgesia.

Characteristic	P for bias^a^	Model	P (I^2^)^b^	Effect Size^c^ (95% CI)	P
**Gender**					
Male	0.52	FEM	0.95 (0.0%)	−0.37 (−0.88–0.13)	0.14
Female	0.99	FEM	0.09 (50.9%)	0.12 (−0.51–0.74)	0.72
*Overall significance test among subgroups*					0.25
**Injury model**					
Contusion	0.99	FEM	0.52 (0.0%)	0.31 (−0.28–0.90)	0.30
Clip compression	0.99	FEM	0.99 (0.0%)	−0.53 (−1.30–0.25)	0.18
Photochemical	0.56	FEM	0.99 (0.0%)	−0.89 (−1.93–0.14)	0.09
Hemisection	0.28	FEM	0.93 (0.0%)	−0.36 (−1.24–0.53)	0.43
Transection	0.80	FEM	0.08 (66.7%)	0.16 (−0.48–0.79)	0.63
*Overall significance test among subgroups*					0.70
**Location of injury**					
Cervical	NA	NA	NA	NA	NA
Thoracic	0.49	FEM	0.16 (33.9%)	−0.08 (−0.51–0.36)	0.72
*Overall significance test among subgroups*					NA
**Severity of injury**					
Moderate	0.44	FEM	0.16 (39.4%)	−0.12 (−0.68–0.43)	0.69
Severe	0.58	FEM	0.28 (22.3%)	−0.03 (−0.62–0.56)	0.92
*Overall significance test among subgroups*					0.85
**OEC derivation origin**					
Bulb	0.56	FEM	0.55 (0.0%)	0.01 (−0.47–0.50)	0.22
Mucosa	0.99	FEM	0.17 (33.6%)	−0.38 (−0.99–0.22)	0.96
*Overall significance test among subgroups*					0.42
**Intervention phase** ^**d**^					
Acute	0.48	FEM	0.23 (26.0%)	−0.08 (−0.53–0.37)	0.73
Subacute	0.99	FEM	0.12 (58.9%)	−0.07 (−1.09–0.95)	0.89
*Overall significance test among subgroups*					0.97
**Graft type**					
Allogeneic	0.71	FEM	0.11 (42.0%)	−0.05 (−0.53–0.44)	0.85
Xenogeneic	0.99	REM	0.76 (0.0%)	−0.24 (−1.02–0.53)	0.54
*Overall significance test among subgroups*					0.71
**Donor species**					
Mice	0.49	FEM	0.07 (50.9%)	−0.26 (−0.92–0.40)	0.97
Rat	0.17	FEM	0.95 (0.0%)	−0.01 (−0.56–0.53)	0.43
*Overall significance test among subgroups*					0.61
**Use of antibiotic**					
No	0.48	FEM	0.30 (17.8%)	−0.19 (−0.66–0.27)	0.42
Yes	0.14	FEM	0.13 (50.9%)	0.13 (−0.64–0.91)	0.74
*Overall significance test among subgroups*					0.50
**Use of immunosuppressive agents**					
No	0.97	FEM	0.08 (60.9%)	0.08 (−0.88–1.04)	0.87
Yes	0.98	FEM	0.41 (0.5%)	−0.17 (−0.56–0.22)	0.40
*Overall significance test among subgroups*					0.60
**Blinding of observer**					
No	0.30	FEM	0.58 (0.0%)	0.11 (−0.38–0.60)	0.44
Yes	0.96	FEM	0.09 (54.4%)	−0.26 (−0.92–0.40)	0.67
*Overall significance test among subgroups*					0.40
**Follow up period**					
<8 weeks	0.54	FEM	0.44 (0.0%)	0.13 (−0.26–0.51)	0.52
≥8 weeks	0.99	FEM	0.58 (0.0%)	−0.66 (−1.28–0.04)	0.04
*Overall significance test among subgroups*					0.07

^a^Publication bias based on Begg’s and Egger’s test; ^b^Heterogeneity among studies; ^c^Standardized mean difference; ^d^Acute: immediately after injury, Subacute: 2–10 days after injury; FEM: fixed effect model, CI: confidence interval; NA: not applicable because of low number of included studies.

#### Efficacy of OEC transplantation on spinal cord injury induced allodynia

Four articles were found in the literature evaluating the effects of OEC transplantation on allodynia^21,25,29,50^. Evaluation of these studies found no significant relation between OEC transplantation and allodynia (Pooled SMD = 0.54; 95% confidence interval: −0.80–1.87; p = 0.43; I^2^ = 86.30%) (Fig. 3). This section also had no publication bias (Coefficient = 11.7; 95% confidence interval: −1.32–24.68 p = 0.07). Although a significant heterogeneity was observed between the studies, subgroup analysis could not be performed due to the small number of articles.

## Discussion

Findings of the present study showed that OEC transplantation significantly improves motor function recovery in animals’ post-SCI. The observed efficacy was affected by the treatment protocol and it was found to be higher when the lesion was in the thoracic region, an allogeneic transplant was used and the cells were derived from rats. Although transplantation of these cells had no significant effect on allodynia in the animals, longer follow-ups were able to reveal that it can lead to aggravation of hyperalgesia.

For the first time, this meta-analysis evaluated the effects of OEC transplantation on neuropathic pain. Among the available literature, a few clinical studies have reported that OEC transplantation does not significantly affect neuropathic pain in subjects with SCI^58^, while others have shown a significant improvement in pain after this treatment^59^. This discrepancy could be attributed to the difference in follow-up periods. For instance, in their study with a follow-up period of 8 weeks, Tabakow *et al*. found a significant improvement in neuropathic pain after OEC transplantation^58^, while Zheng *et al*. reported no significant improvement in their subjects after a 12 month follow-up period^59^. The present study also showed that longer follow-up periods were associated with reports of OEC transplantation negatively affecting neuropathic pain post-SCI. Hence, further investigations are required to reach a consensus on this subject.

The overall results of the present study regarding the effects of OEC transplantation on motor function recovery were congruent with the two previous meta-analyses performed; the study conducted by Liu *et al*. that included six animal surveys and reported that OEC transplantation can improve functional recovery^22^, and the study conducted by Watzlawick *et al*. which confirmed these results^23^. The results of our study cannot be further compared to Liu *et al*.’s since they did not perform subgroup analysis on their data. On the other hand, Watzlawick *et al*. carried out subgroup analysis, the results of which were incompatible with that of the present survey. These authors found that OEC transplantation performed immediately after photochemically induced injuries with doses of 1.8 × 10^5^ to 1.5 × 10^5^ is associated with better motor function recovery. Moreover, the OEC transplantation was found to be more effective when the cells are fractionated, derived from the olfactory bulb and injected into the rostral-caudal parenchyma. On the contrary, in the present study allogeneic transplants, treatment of thoracic lesions and OECs acquired from rats were associated with greater improvements in motor function. These discrepancies might be due to differences in inclusion and exclusion criteria of the studies. For instance, in the present study using directed forelimb reaching test, olfactory tissue blocks and combination protocols were considered as exclusion criteria to decrease heterogeneity of the included studies; while Watzlawick *et al*. included surveys with these conditions. Furthermore, based on the current guidelines, performing subgroup analyses and multiple meta-regressions in a meta-analysis can lead to a bias, known as data dredging^60^. Accordingly, we performed subgroup analysis only for the most important factors affecting the efficacy of OEC transplantation on SCI complications. This might be the reason that meta-regression yielded more significant factors in the Watzlawick *et al*.’s study.

The optimum cellular dose for OEC transplantation in SCI was reported to be 1.8 × 10^5^ to 1.5 × 10^5^ by Watzlawick *et al*., while no such relation was observed in the present study which could be due to the difference in definition of cellular dose in the two studies. Watzlawick *et al*. included crude numbers of transplanted cells into their analysis while the cellular dose in our study referred to the crude numbers standardized for the weight of the animals. Since different animal species (mice and rat) were evaluated in the included studies, this standardization seems to be of utmost significance; a certain dose (crude dose) of transplanted cells in mice might be considered a high dose, while the same amount in rats might be regarded as moderate or even low dose^23^.

In the present study, extensive search in electronic databases, contacting authors of the articles and manual search yielded the extreme number of articles and included non-indexed literature. This method led to inclusion of 40 articles and 45 experiments in present study. On this basis, data from 933 animals including 464 controls and 469 treated animals were analyzed. Lack of publication bias is another advantage of this study. Although a significant heterogeneity was observed in evaluation of motor function recovery, the extensive search provided homogeneity in assessment of hyperalgesia. The limitation of heterogeneity in the included studies was tackled by performing subgroup analyses. Not blinding the researchers in some of the included studies was another limitation of the present survey which might have subjected our results to bias. However, since blinding status had no significant relation with efficacy of OEC transplantation in subgroup analyses, it seems that the bias is at its minimum level. Another factor that could be a potential source of heterogeneity is the purity of transplanted OECs. Although most of the included articles have declared application of “high purity” OECs, few have actually provided evidence for their claim.

## Conclusion

The present meta-analysis showed that OEC transplantation significantly improves motor function recovery of the animals after SCI. It seems that this treatment is most effective on motor function recovery, when it is used in a thoracic SCI rather than a cervical injury, when an allogeneic transplant is performed and when the cells are derived from rats. Although the treatment does not affect allodynia, longer follow-ups reveal relative aggravation of hyperalgesia following OEC transplantations. Since findings of clinical studies regarding the relation between OEC transplantation and neuropathic pain are inconsistent and aggravation of pain is one of the limitations for using this treatment, further studies with longer follow-up periods should be conducted to assess the effects of OEC transplantation on the severity of neuropathic pain. Finally, the effects of OEC transplantation should be interpreted with caution since the treatment may not be beneficial in every setting. Accordingly, further investigations are required to determine the subgroups of patients and the specific settings that benefit the most from this treatment.

## Methods

The study was conducted in accordance to the Preferred Reporting Items for Systematic Reviews and Meta-Analyses (PRISMA) guidelines^61^.

### Search strategy

We searched several databases including Web of Science (BIOSIS), Medline (via PubMed), Scopus, Embase (via OvidSP), and ProQuest from the beginning of the year 1944 to the end of 2015. Keywords related to “olfactory ensheathing cells” combined with terms related to “spinal cord injury” were used in the search. The combined keywords in the three databases of Embase, Medline and Scopus are presented in Table 4. The method through which these keywords were selected and combined is presented in previous surveys^62,63^.

**Table 4:** Keywords used for search in Medline, Embase, and Scopus databases.

Database	Search terms
Medline (PubMed)	“olfactory ensheathing cell*“[mesh] OR “olfactory bulb cell*“[mesh] OR “Olfactory ensheathing glia”[mesh] OR “ensheathing cell*“[tiab] OR “Olfactory Cortex cell*“[tiab] OR “olfactory cell*“[tiab] OR “olfactory bulb‐ensheathing cell line”[tiab] OR “olfactory nerve ensheathing cells”[tiab] OR “ensheathing cell*“[tiab] OR “Olfactory ensheathing glia*“[tiab]] OR “olfactory schwann cell*“[tiab] OR “schwann cells of the olfactory nerve”[tiab] AND “Spinal cord injuries”[MeSH] OR “Spinal cord contusion”[tiab] OR “Spinal cord transection”[tiab] OR “Injured spinal cord” [tiab] OR “Spinal Cord Trauma”[tiab] OR “Spinal cord Hemisection”[tiab] OR “Spinal compression”[tiab] OR “Traumatic Myelopath*“[tiab] OR “Spinal Cord Laceratio*“[tiab] OR “Post-Traumatic Myelopath*“[tiab]
EMBASE (OvidSP)	exp olfactory ensheathing cell/OR (olfactory ensheathing cell$ OR olfactory bulb cell OR Olfactory ensheathing$ glia OR ensheathing cell$ OR Olfactory Cortex cell$ OR olfactory cell$ OR olfactory bulb ensheathing cell line OR olfactory nerve ensheathing cells OR olfactory schwann cell$“ OR schwann cells of the olfactory nerve).ti,ab. AND exp Spinal cord injuries/OR (Spinal cord contusion OR Spinal cord transection OR Injured spinal cord OR Spinal Cord Traum$ OR Spinal cord Hemisection OR Spinal compression OR Spinal Cord Laceratio$).ti,ab.
SCOPUS	((TITLE-ABS-KEY (olfactory ensheathing cell) OR TITLE-ABS-KEY (olfactory bulb cell) OR TITLE-ABS-KEY (olfactory ensheathing glia) OR TITLE-ABS-KEY (ensheathing cell) OR TITLE-ABS-KEY (olfactory cortex cell) OR TITLE-ABS-KEY (olfactory cell) OR TITLE-ABS-KEY (olfactory bulb ensheathing cell line) OR TITLE-ABS-KEY (olfactory nerve ensheathing cells) OR TITLE-ABS-KEY (olfactory schwann cell))) OR TITLE-ABS-KEY (schwann cells of the olfactory nerve))) AND ((TITLE-ABS-KEY (spinal cord injuries) OR TITLE-ABS-KEY (spinal cord injury) OR TITLE-ABS-KEY (spinal cord transection) OR TITLE-ABS-KEY (spinal cord hemisection) OR TITLE-ABS-KEY (injured spinal cord) OR TITLE-ABS-KEY (spinal cord trauma) OR TITLE-ABS-KEY (spinal compression) OR TITLE-ABS-KEY (spinal cord contusion)))

Along with the conducted systematic search, manual search was performed to yield further articles and grey literature. The search technique for grey literature has been described in the previous meta-analyses conducted by the authors^11,62–68^. Briefly, search in Google Scholar and Google Search Engine was performed based on the keywords related to the study’s questions. Moreover, the authors of articles with similar aims and methods were contacted via email and theses were searched in the ProQuest database. Finally, in order to find additional articles, bibliographies of related articles were reviewed and manual-searching of highly focused journals was carried out. Four more articles were found via this method.

### Eligibility criteria

All the controlled animal experiments published from the beginning of the year 1944 until the end of 2015 which evaluated the effects of OEC transplantation on recovery of motor function, hyperalgesia and allodynia after SCI were included in the present study. No linguistic limitations were applied. Inclusion criteria were as follows: 1) *in vivo* animal experiments regardless of the age, gender or species of included subjects; 2) induction of SCI based on standard models of contusion, compression, hemisection, transection and photochemical injury; 3) moderate and severe injuries. Exclusion criteria included any modifications of transplanted cells, application of combined therapy methods, transplantation of olfactory tissue blocks, follow-up of less than 4 weeks, evaluation of the outcome according to unstandardized behavioral tests and lack of a control group (spinal cord injured animals, treated by saline or vehicle).

### Data extraction and quality assessment

Search, summarization, data gathering and assessment were carried out by two independent reviewers. Any disagreements were solved through discussion with a third researcher (89% agreement). Data gathering was performed based on an online checklist designed according to PRISMA guidelines. After elimination of repetitive studies, initial screening was carried out and potentially eligible studies were selected, their full-texts were studied and data were extracted from the ones that met inclusion and exclusion criteria. Extracted data are presented in Table 1 which includes characteristics of evaluated animals, treatment protocol, follow-up duration, outcome and possible biases. The method proposed by Sistrom and Mergo for data extraction from charts was utilized as needed^69^. If the outcome was assessed multiple times during a study, the last measurements were included. If data were not presented in the article, the authors were contacted and in cases of no response, two reminders were sent with one week intervals. If the corresponding author did not respond, social networks such as LinkedIn and ResearchGate were used to make contact with other authors of the article. Finally, quality assessment of the articles was carried out based on the 19-item checklist designed by Yousefifard *et al*.^62^.

### Statistical analysis

All the analyses were performed by the STATA 11.0 software. Data were summarized as means and standard deviations, and standardized mean differences (SMD) were computed with a 95% confidence interval according to Hedges’ g. Eventually, a pooled effect size was calculated. Publication bias was evaluated using Egger’s and Begg’s tests^70^. Interstudy heterogeneity was considered using Chi-squared and I^2^ tests. If this test provided evidence of heterogeneity (p value less than 0.1 or an I^2^ greater than 50%), random effect model was applied, otherwise we used fixed effect model. In random effect analyses, 95% predictive intervals were calculated to illustrate the degree of heterogeneity and to predict true treatment effect in an individual study^71,72^.

Subgroup analysis was conducted to evaluate the differences between different treatment protocols in efficacy of OEC transplantation on recovery of motor function and sensory status of the subjects. Statistical significance level was considered at a P value of less than 0.05.

### Data Availability

The datasets generated during this meta-analysis could be shared by the corresponding author on reasonable request.
